# Hysterical Paralysis

**Published:** 1910-02-12

**Authors:** Grainger Stewart


### Hospital Clinics.

### HYSTERICAL PARALYSIS.

By GRAINGER STEWART, M.D.

(A lecture delivered at the National Hospital for the Paralysed and Epileptic, Queen Square, W.C., on Friday, December 17, 1909.)

Gentlemen,—I want to talk to you to-day about some cases of hysterical paralysis. Hysteria is a condition which, from the very multiplicity of its symptoms and its manifestations, has always presented some difficulty; sometimes in the diagnosis, but more seriously in regard to the interpretation of its cause. A very interesting book has been published in English by Dr. Père Janet; and if in what I have to say I follow somewhat closely that book, it is because I think it contains the best explanation we have, so far, of hysteria. Janet holds that hysteria is practically identical with somnambulism. This latter is the condition in which a person, apparently in sleep, walks about, talks, and acts. The actions of such people seem to follow out some special idea, yet the patients are quite unconscious of their surroundings, even when spoken to or touched.

I will now rapidly pass to what occurs in certain cases of hysteria. In some you see the manifestations owing to one special idea. The patient, perhaps, has a fright; may be at the death-bed of a friend; or may see a murder or a suicide. Though at the actual occurrence the person may seem calm and collected, yet later she falls into a hysterical state, in which she appears to be going through the scene which caused her fright. Janet cites one case of a woman who was called to see her sick niece. When she arrived she found the niece had jumped out of the window and was lying dead. She appeared collected, but after the funeral began to have attacks of hysteria. Later she began to talk of this girl and tell what a good life she had led, and how she had admired her. She would then wander about and open the window, and if she had not been watched she also might have ended her life. She would suddenly awake from such states, and have no recollection of what had happened, and at ordinary times the prime cause of the attack is not present to the mind.

### Somnambulism.

Somnambulism may start either suddenly or gradually. In the sudden cases the patient half faints, and then passes into a condition in which she is oblivious to the outside world. When the onset is gradual, her attention wanders, she does not answer when spoken to, and she dreams off into this separated condition. In the cases where one idea develops, you find the manifestation is very intense, so that everything else is cut off for the time being. In the book I have mentioned there is quoted the case of a woman who had a fright at a zoological gardens by a lion or a tiger. In her attacks she apparently imagined herself to be that beast; she would go down on all fours, and growl and prance about; and she even went the length of getting photographs of human beings and pretending to eat them. On recovery she had no recollection of the occurrence, nor was her visit to the zoological gardens in her mind. Owing to this abstraction, people in this condition who have lost a relative are sometimes said to be callous and indifferent, whereas there is no doubt they feel most intensely. I have gone thus fully into this aspect because it is upon this basis that Janet seeks to explain the various phenomena in hysteria.

Before coming to definite cases, let us pass for a minute to some of these other strange hysterical cases which one occasionally reads of in the papers, the sort of case which I think is frequently overlooked, cases in which, on account of some shock or fright, the person gets so possessed with an idea that he runs away. Janet cites a man who, when younger, had been a somnambulist. In his business certain charges were made against him, and, though he knew them to be false, and though they were not very serious and he could easily have refuted them, they began, as he was hysterical, gradually to take effect, and eventually he ran away. He remained away eight days and was not traced. And it was found afterwards that in all his wanderings he had been careful to hide his identity, such as by going from a station some distance away, where he was not known. He was brought back to his normal state by someone happening to mention the date, which happened to be the anniversary of his birthday, and then he wondered where he was. From a letter on him his address was found, and he was sent home. Later he had another similar attack. In prolonged attacks there is not the same completeness of isolation from surroundings, otherwise precautions to escape detection could not be taken. Often when asleep this man would mutter concerning the charges, and he would sometimes be heard to be making agonised pleadings against injustice.

### Dual Personality.

There are still more extraordinary cases recorded, in which people have had, apparently, what is known as dual personality. In such people there is a normal state and an abnormal state. In the one the character may be somewhat melancholy and timid, while in the other it is cheerful and bright. In either of these states there is no knowledge of the other. In a number of cases the condition has been carefully worked out; and psychologists have tested their reactions to see what they would be in similar circumstances in the two states.

Now let us pass to some of the symptoms which are met with in hysteria. First, the convulsive attacks. You may see almost every variety of hysterical fit, and almost every grade. By watching the patients you can sometimes recognise the sort of idea which is acting in their mind. Sometimes you may see them in an attitude of adoration; others may be seen trying to push somebody away. The distinction between an epileptic and a hysterical attack is one which you may at any time be called upon to make. I have not time to enter fully into this, but I may point out some matters of interest. In epilepsy there may or there may not be an aura; and when there is one, it is usually the same in every fit. If a person with epigastric aura has *petit mal*, that is really the aura of the major attack. In an epileptic fit the patient becomes convulsed; he may, or may not, know that something is going to happen. In many cases, but not all, the patient utters a cry and then falls down unconscious. Of course, injury may result from the fall. Usually in an epileptic fit, though it may be bilateral, the cortical discharge is greater on one side of the brain than on the other. The motor part of an epileptic fit is, first of all, a tonic stage, in which the patient becomes more or less rigid; and as that passes off there is a very fine tremor, which becomes of less frequent occurrence, more extensive in range, and then becomes definitely clonic. It ends by one or two severe jerks, then he breathes heavily, and feels more or less stupil and dull. If the patient becomes unconscious, the pupils will be found to be inactive to light.

### The Reflexes.

I spoke of the fit affecting one side more than the other; therefore, you find the head and eyes are deviated to one side, *i.e.* to the side opposite to the cerebral hemisphere which is discharging the more strongly. And if you examine the patient when the fit is over you will find certain other things. As a rule the temperature is raised, and you will find that reflex changes are present as in organic disease. Shortly after the fit you may find no deep reflex, but soon the reflexes are greatly exaggerated on both sides, more especially on the affected side. You may not get a plantar response at all, and when this response does return it is at first extensor in type—the great toe moves upward, but a few minutes later you may find it has returned to normal flexion. It may not be permanent; it may pass off in a few moments or in an hour or two. If it remains longer, the probability is that there is organic disease of the brain pressing on the opposite hemisphere which is causing these symptoms. In regard to passing water: if they pass water it is in the tonic stage. All the muscles are then rigid and press on the bladder region. And if they have strong clonic movements they may dislocate their shoulder, or perhaps their jaw. In the clonic stage the chief action is tongue biting.

In hysteria there may be a warning, or there may not. As a rule, there is some definite cause; it may be some emotion or nervousness. It may be due to the patient's temperament, to her craving for sympathy; when an idea overcomes them they pass into the hysterical state. It is practically impossible to make a person have an epileptic fit when you want to, but you can do that with a hysterical patient. I do not think that consciousness is ever completely lost in hysteria, though apparently they are oblivious to external things. If you touch the cornea in an attack there is mostly some response, and I believe the pupils are never inactive. These people may utter a cry or a series of shrieks; they may even talk and call out names. With regard to their movements, they are not at first tonic and then clonic, they are more or less co-ordinated; often they are so marked that you can follow the sort of idea that they express. I shall show you soon a girl whose fits were brought on by a definite cause. The movements may last for a long time, and they are very irregular; one may see the patient making certain movements one moment, then keeping still, then suddenly jumping up again. You do not see that in epilepsy. In hysteria the patient, while in bed, may arch her back, resting only on her heels and head, and next moment may make the face almost touch the knees. In hysteria, too, if you interfere the patient resists you; frequently she will try to bite those who are attending to her. She will not bite her tongue, but will often bite her lips, and will often pass her water immediately after a fit. An epileptic fit rarely lasts more than two or three minutes; a hysterical attack may last twenty minutes, half an hour, or hours. What happens when the fit is over? The hysterical patient wakes up suddenly and at once realises where she is, but has very little idea of what happened during the fit. Where there is one definite idea accounting for the whole seizure the patient may not only not know what happened during the fit, but may forget the original cause of it. In the slighter cases patients know little of what happened, but the cause is still in their mind.

### An Illustrative Case.

The girl I shall show you first is 15¼ years of age; and as a child she suffered from nocturnal enuresis. She had been noticed to be a nervous child. She had never shown any obvious sign of hysteria, but I cannot ascertain whether she ever walked in her sleep. In August she was at home alone with a little child whom she was looking after: her father and mother were out. A man knocked at the door, and the child ran to open it. The man asked "Is the governor in?" The child replied "No." The girl, not liking the sound of his voice, went to the door and said, "He is not in"; as she did not want him to come in. The man evidently did not believe her, and started to frighten her. Two days afterwards she had a severe hysterical fit, and she has had a great many more, though since she has come into hospital they have practically ceased. I shall ask her to tell you what it is she feels before the fits come on. The man's name is Charley, and it has been found that the mere mention of that name will bring on a fit. She tells us that before a fit there comes a kind of black mist before her eyes—it was dark when the man came—and then she knows nothing more. After the attack she has severe frontal headache. During the fit she seems to be trying to keep someone off.

I want now to pass to another form of hysterical manifestation, namely, trance. In this, the patient passes into what seems a very light sleep; the breathing is very light, the colour is waxy, you may not be able to feel the pulse at the wrist, and the temperature becomes lowered. But as a rule you will be able to see some tremulousness about the eyes; you can detect breathing, and with the stethoscope you can hear the heart beats. I have only seen one such case, and I saw her on two occasions. She was a general servant. In the place she had been in before, she had had to sleep with another servant, in the same bed, and this servant died during the night. She aroused the household. Apparently she illustrated two effects: the fit and the agitation; she got the idea that she was dead, and for ten days she remained in a trance. In the majority of people who have hysterical attacks, an attack can be brought on by mentioning what caused the original attack.

### Motor Symptoms.

I now want to consider briefly some of the motor symptoms. I shall not speak of the hysterical tics, which are so common—people who are blinking and grimacing. Remember, you must distinguish between hysterical tics and psychosthenic tics. In hysterical tics the patient may be unaware that the movement is taking place, and so it causes him no distress; and if you draw his attention to it he can probably stop or diminish it. But in psychosthenic tics, the patient experiences great agony, and it has probably developed out of a habit. For instance, Sir Walter Scott, when he was at school, used always to finger with a button when answering questions; and when someone cut the button off he got confused and broke down. If when he was not answering questions he had been fingering the butter it would have been a psychasthenic tic. Patients know they are doing it; they do not want to do it, they struggle against the tendency for a time, and eventually conclude that if only they do it once they will feel better; and when they have yielded the same process goes over again. When they have yielded they suffer remorse or psychic pain, whereas the hysterical do not. In psychasthenic tic, if you distract the attention the tic stops, while if you draw attention to it it becomes more marked.

It is uncommon to get tremors in hysteria, but when they occur they are irregular, coming and going. With regard to the motor system, you may find contractures; it is very difficult to differentiate hysterical contracture from that due to an organic cause. You may find the arm very strongly drawn up, not only through the day, but during sleep; and it may last for months, even years. In hysterical contracture you will find certain signs absent which are present in organic disease, and that there are certain of the stigmata of hysteria.

With regard to hysterical paralysis, I will bring before you a short idea which I think will help you to grasp the occurrence of paralysis in hysteria. In front of the fissure of Rolando there are motor centres, and behind it there are sensory; in front of the motor there are certain specialised centres. There may be loss of the power of writing, owing to a lesion, yet the patient may be able to copy. Dr. Hughlings Jackson divided the fits of epilepsy into three kinds. The first type is in regard to the highest centre, where the idea originates. If you get this highest consciousness cut off, what will happen? The motor and sensory powers are all right. Dr. Jackson held that in the attacks of automatism, what has happened is that owing to some slight discharge affecting the highest centre, it is cut off, and that the patient is acting more or less subconsciously, but he acts well because his motor and sensory symptoms are intact. In the hysterical person the whole attention, during an attack, is concentrated on one idea, and consciousness to the rest of the world is lost. Analogous to the motor centres you may have the psychical centres affected. If the psychical centres are alongside the motor, what will happen? You might quite well get a person with a psychical affection, say, affecting the arm; he can feel his arm, and can move it, but does not will to put it into action. The psychical centre is cut off. So you may say it is a condition in which you get a diminution, loss, or inequality of consciousness.

In a patient with hysteria you will often find hemiplegia, with characters which are more or less those of organic disease; but in hysteria there is not a paralysis of a certain muscle or set of muscles, but of movement.

### Sensory Changes.

With regard to sensory changes, there may be hemiplegia with hemianæsthesia. But if there is a monoplegia in the hand, what do you find? He cannot close or open his hand, and the sensation of the hand also is affected; and it will be found that this sensory loss, although it is more or less limited to the periphery of the limb, is not bounded by a sharp upper limit. I want to show you that the character of the paralysis is really not very unlike what you get in cortical cerebral disease. If you take my supposition that you have corresponding centres to those motor and sensory, you can understand how, in hysteria, if you get consciousness of one of these lost, there may be just those manifestations of the paralysis, sensory or motor, with the rest of the body normal. There may be contractures in hysteria, but, on the other hand, a limb may be flaccid, and in the latter you will generally find there is complete sensory loss; these patients have no idea that they have an arm. Even a person who has had his arm amputated can recall and imagine the arm movements. As a rule, you do not find spasticity in hysteria, and where there are contractures they will generally disappear under an anæsthetic, unless they have existed so long that there are organic changes. In organic cases, even under an anæsthetic, there is stiffness. Here is a man who is suffering from traumatic hysteria. He is 45 years of age, and on October 17 he suffered from two accidents. First, he fell and came down astride a joist, which hit him over the coccyx, causing severe pain. An hour later some wood fell and struck him on his back. His right leg is weaker than its fellow. I can move it for him easily, so there is no spasticity; the limb is flabby and thin through lack of use. When I hold his limb and ask him to bend it down he can hardly do it at all, neither can he elevate it. You never find that in organic disease, because if he could not move it then it would drop from sheer weakness. To sustain a limb in the position in which it is placed needs power, but there is not the will. His knee jerk is very brisk. I cannot elicit clonus. The plantar is flexor in type. You see he has loss of sensation in the limb up to just above the knee. The sensory loss may be to all forms or only to some. As a rule, sensation to cotton wool and to ordinary touch are more completely gone than other forms. He has not had any trouble with his water. He has got no organic disease at all. Perhaps I got a flexor plantar response because he has some anæsthesia of the sole of his foot. This man did not know until he came into the hospital that he had loss of sensation in his leg, and most of the people with hysteria are unaware that they do not feel; it is very rare for a hysterical person to have numbness and tingling, whereas subjects of disseminated sclerosis often do. This man's idea is that his back was injured, and paralysis of his leg resulted. He will get quite well.

As you are aware, there may be concentric loss of the fields of vision in hysteria: he sees only with central vision. An interesting point is, that where the hysteria comes on as the result of fire, you find the vision is contracted to central except for a flame, so that if you hold a lighted candle almost out of the normal field the patient sees it.

This patient whom I show you next walks with her foot inverted, and she tends to drag it. I can straighten it, and she does not feel pain. Her knee jerks are brisk, she has not got ankle clonus. There is a typical flexor response in the great toe. She has almost recovered.

In your general practice, not only in cases of nervous disease, but in every form of disease, if you watch carefully you will see a great deal of hysteria, and it will explain many things which would otherwise puzzle you. Remember the probability of a certain amount of hysteria being present. It is the recognition of this that makes some men more successful in practice than others. Much depends on being able to get into the minds of patients that they are going to get better. The subject is far too large a one for me to expect to do justice to it in an hour's lecture, but I would strongly advise you to read Janet's book; you may not agree with all he says; you may disagree with parts of it, but I am sure you will find it well worth perusal.

